# The influence of polycystic ovary syndrome on abortion rate after in vitro fertilization/intracytoplasmic sperm injection fresh cycle pregnancy

**DOI:** 10.1038/s41598-023-32988-5

**Published:** 2023-04-12

**Authors:** Qian Dou, Li-ying Ma, Peng-fen Li, Xiao-ting Xu, Guo Yu, Dan Zhang, Yun-gai Xiang, Li Tan

**Affiliations:** Department of Reproductive Medicine, The Second Affiliated Hospital of Zhengzhou University, No. 2, Jingba Road, Zhengzhou, 450014 China

**Keywords:** Diseases, Risk factors

## Abstract

There are many reports on clinical pregnancy outcomes in polycystic ovary syndrome (PCOS) patients receiving vitro fertilization (IVF) or intracytoplasmic sperm injection (ICSI), but little research about abortion has been done and there is a debate on whether the abortion risk increases in PCOS patients receiving IVF/ICSI. Therefore, the aim of this study was to investigated the abortion in PCOS patients. Clinical data of 12055 IVF/ICSI fresh cycles performed in our hospital from January 2015 to December 2020 were collected. Based on the Rotterdam diagnostic criteria of PCOS and after propensity score matching (PSM) for baseline data of clinical pregnancy cycles, matched 599 PCOS (PCOS group) and Non-PCOS (non-PCOS group) cycles were obtained. Abortion and abortion-related outcomes were compared between the two groups. Risk factors for late abortion in twins were analyzed using binary Logistics regression. Post-PSM data showed that the late abortion rate was significantly higher in the PCOS group than in the non-PCOS group only in twin pregnancy (9.50% vs. 3.96%, OR: 2.55, 95%CI 1.10–5.89). There were no statistical differences in other pregnancy outcomes. The etiological distribution for late abortion were not statistically different between the two groups in both singletons and twins. Logistics regression indicated that PCOS and obesity [pregnancy-assisted body mass index (BMI) ≥ 28] were risk factors for late abortion in twin pregnancy. In twin pregnancy, PCOS and obese patients are more likely to have late abortion. In twin pregnancy, the late abortion risk significantly increased in the PCOS patients as compared with non-PCOS patients (OR: 2.59, 95%CI 1.11–6.03, *P *< 0.05), as well as in the patients with obesity (BMI ≥ 28) as compared with the patients with normal BMI (OR: 4.17, 95%CI 1.59–10.90, *P *< 0.05). PCOS does not significantly affect early and overall late abortion rates after IVF/ICSI fresh cycle pregnancy.

## Introduction

Polycystic ovary syndrome (PCOS), one of the most common endocrine and metabolic disorders, affects 8%-13% of women at childbearing age^1,2^, and is a common cause of infertility for women^1,3,4^. Based on international diagnostic criteria, namely "Rotterdam criteria", PCOS is characterized by sparse ovulation or anovulation, clinical or biochemical hyperandrogenemia, and polycystic ovary^5^. Some patients with PCOS require in vitro fertilization/intracytoplasmic sperm injection (IVF/ICSI) when they are not able to be pregnant after ovulation induction, and adjusting lifestyle or menstrual cycle. PCOS pathophysiological features, including hyperandrogenemia, obesity, insulin resistance and metabolic disorder may lead to adverse pregnancy outcomes, such as elevated abortion, pregnancy complications and premature birth rates^6–9^. The focus of reproductive research has been changing from improving the success rate of assisted reproductive technology (ART) to ensuring the safety for both mother and fetus. Although it has been reported that the risk of adverse pregnancy outcomes increases in PCOS patients receiving ART^10–13^, there is no consensus on abortion of PCOS patients during gestational period. In China, late abortion is defined as the abortion occurring between 12 and 28 gestational weeks. Although the later abortion rate is not high, the psychological harm induced by the late abortion to pregnant women is more serious, especially for the women with high expectations of pregnancy. There is a debate on whether the abortion risk increases in PCOS patients receiving IVF/ICSI^9,11–13^. In this study, we collected clinical data of PCOS patients receiving IVF/ICSI in our hospital from January 2015 to December 2020, mainly observed abortion and analyzed the risk factors of late abortion after controlling confounding by propensity score matching (PSM). This study provided a guidance for the management of PCOS patients during gestational period.

## Materials and methods

All study methods were approved by the Ethics Committee of the Second Affiliates Hospital of Zhengzhou University (2022162), and were performed in accordance with relevant guidelines and regulations. All the subjects enrolled into the study gave written informed consent to participate.

### Subjects

These data were from the electronic medical record system of the Department of Reproductive Medicine, the Second Affiliated Hospital of Zhengzhou University. The clinical data of all patients receiving IVF/ICSI in the Department of Reproductive Medicine, the Second Affiliated Hospital of Zhengzhou University from January 2015 to December 2020 were collected, and then were retrospectively analyzed.

In this study, PCOS was diagnosed according to "Rotterdam criteria"^5^. The "Rotterdam criteria" are that the patients may be diagnosed with PCOS, when the patients have 2 of the 3 items including (1) sparse ovulation or anovulation (menstrual cycle ≥ 35 days or duration difference between consecutive menstrual cycles > 10 days), (2) hyperandrogenemia (total serum testosterone > 0.75 ng/ml, androstenedione > 3.9 ng/ml or dehydroepiandrosterone [DHEAS] > 3.14 ng/ml) or hyperandrogenemia-related clinical manifestations (Ferriman-Gallwey score > 6 and/or severe acne and alopecia); (3) polycystic ovary showed by ultrasound (at least one ovarian volume > 10 ml or more than 12 2–9 mm antral follicles), as well as other diseases that may cause hyperandrogenemia and/or abnormal ovulation are excluded.

Exclusion criteria were (1) cycles of donor semen or oocytes; (2) uterine malformation such as unicornuate uterus, bicornate uterus and septate uterus; (3) abnormal chromosomal karyotype in one or both of the couple; (4) endocrine disorders such as thyroid dysfunction and hyperprolactinemia; (5) two or more spontaneous abortions occurring within 28 gestational weeks, or 3 or more high-quality embryo transfer failures; (6) tuberculosis of reproductive system or other systemic diseases; and (7) cycles with missing key data.

### Treatment

Before treatment, venous blood was taken from each patient for determining follicle-stimulating hormone (FSH), luteinizing hormone (LH), estrogen (E_2_)**,** progesterone, testosterone and Anti-Mullerian hormone (AMH), and each patient underwent transvaginal ultrasound to obtain Anti-Mullerian hormone (AFC). Appropriate ovarian stimulation protocol was selected according to patient's factors such as age, ovarian reserve and body mass index (BMI)^14,15^. In this study, three ovarian stimulation protocols were applied including long gonadotrophin-releasing hormone agonist (GnRH-a) protocol, prolong GnRH-a protocol and GnRH antagonist (GnRH-ant) protocol. The GnRH-ant protocol was used as the first choice for PCOS patients. The Gn used in this study was recombinant human follicle-stimulating hormone (Gonal-F, Serono, Switzerland) or urofollitropin (Lishenbo, Livzon Pharmaceutical Group Inc, China). The starting dose of Gn was based on patients age, AFC, basal FSH and BMI. The dosage of gonadotropin (Gn) was adjusted according to follicular development and serum hormone levels. When the diameter was more than 18 mm in at least two ovarian follicles or the diameter was more than 16 mm in 50% of all ovarian follicles, 10,000 IU of human chorionic gonadotropin (HCG, Lizhu Pharmaceutical, China) was given. Thirty-six or 38 h later, oocyte retrieval was performed under ultrasonic guidance. IVF or ICSI was selected according to sperm quality, and then was performed at 39–40 h after HCG injection. After 16–18 h of fertilization, pronuclei were observed, and 2 pronuclei were regarded as normal fertilization. Based on patient's endometrium, hormone level, and clinical manifestations, it was determined whether embryo transfer was performed in 3–5 days after oocyte retrieval. On HCG day, if the patient's endometrial thickness < 7 mm, number of oocyte retrieval > 15, blood estradiol level > 4000 pg/ml, ovarian diameter ≥ 7 cm, we recommended whole embryo cryopreservation. For the patients suitable to fresh embryo transfer, the number of embryos transferred was not more than 2, and the remaining available embryos were vitrified for cryopreservation. The patients received conventional luteal supportive therapy after IVF or ICSI.

### Outcome measures

The aim of this study was to investigate the abortion in PCOS patients receiving IVF, so only abortion and abortion-related outcomes were observed. The outcome measures of this study included early abortion rate, overall late abortion rate, late abortion rate in singleton pregnancy, late abortion rate in twin pregnancy, multiple pregnancy rate, ectopic pregnancy rate, stillbirth rate, final live birth ratio and pregnancy complication rate.

On the fourteenth day after embryo transfer, serum HCG ≥ 20 IU/L was regarded as biochemical pregnancy. It was diagnosed as clinical pregnancy that B-mode ultrasound showed gestation sac 30 days after embryo transfer. The abortion occurring between 4 and 12 gestational weeks was defined as early abortion. It was regarded as late abortion when the abortion occurred between 12 and 28 gestational weeks and the fetal weight was less than 1000 g. It was defined as live birth if the fetus with 28 gestational weeks or more had heartbeat, breathing, funic pulse or voluntary muscle contraction after delivery. The cycle with at least one living fetus was called as live birth cycle. Gestational complications mainly include gestational hypertension and diabetes mellitus. Follow-up lasted until October 2021.

$${\text{Early}}\;{\text{abortion}}\;{\text{rate}} = {\text{Number}}\;{\text{of}}\;{\text{early}}\;{\text{abortion}}\;{\text{cycles}}/{\text{Number}}\;{\text{of}}\;{\text{clinical}}\;{\text{pregnancy}}\;{\text{cycles}} \times 100\%$$

$${\text{Overall}}\;{\text{late}}\;{\text{abortion}}\;{\text{rate}} = {\text{Number}}\;{\text{of}}\;{\text{late}}\;{\text{abortion}}\;{\text{cycles}}/{\text{Number}}\;{\text{of}}\;{\text{ongoing}}\;{\text{pregnancy}}\;{\text{cycles}} \times 100\%$$

$${\text{Late}}\;{\text{abortion}}\;{\text{rate}}\;{\text{in}}\;{\text{twins}} = {\text{Number}}\;{\text{of}}\;{\text{late}}\;{\text{abortion}}\;{\text{cycles}}\;{\text{in}}\;{\text{twins}}/{\text{number}}\;{\text{of}}\;{\text{ongoing}}\;{\text{pregnancy}}\;{\text{cycles}}\;{\text{in}}\;{\text{twins}} \times {1}00\%$$

$${\text{Multiple}}\;{\text{pregnancy}}\;{\text{rate}} = {\text{Number}}\;{\text{of}}\;{\text{multiple}}\;{\text{pregnancy}}\;{\text{cycles}}/{\text{number}}\;{\text{of}}\;{\text{clinical}}\;{\text{pregnancy}}\;{\text{cycles}} \times {1}00\%$$

$${\text{Ectopic}}\;{\text{pregnancy}}\;{\text{rate}} = {\text{number}}\;{\text{of}}\;{\text{ectopic}}\;{\text{pregnancy}}/{\text{number}}\;{\text{of}}\;{\text{clinical}}\;{\text{pregnancy}}\;{\text{cycles}} \times {1}00\%$$

$${\text{Stillbirth}}\;{\text{rate}} = {\text{Number}}\;{\text{of}}\;{\text{all}}\;{\text{dead}}\;{\text{fetus}}\;{\text{at}}\;{\text{childbirth}}/{\text{number}}\;{\text{of}}\;{\text{delivery}}\;{\text{cycles}} \times {1}00\%$$

$${\text{Final}}\;{\text{live birth}}\;{\text{ratio}} = {\text{Number}}\;{\text{of}}\;{\text{live}}\;{\text{birth}}\;{\text{cycles}}/{\text{number}}\;{\text{of}}\;{\text{clinical}}\;{\text{pregnancy}}\;{\text{cycles}} \times {1}00\%$$

$${\text{Gestational}}\;{\text{complication}}\;{\text{rate}} = {\text{Number}}\;{\text{of}}\;{\text{gestational}}\;{\text{complication}}\;{\text{cycles}}/{\text{number}}\;{\text{of}}\;{\text{clinical}}\;{\text{pregnancy}}\;{\text{cycles}} \times {1}00\%$$

### Statistical analysis

The PASS11.0 software was used to perform a power analysis, estimating the sample size. According to the previous study^16^, the late abortion rate in PCOS group was assumed to be 6% and the late abortion rate in non-PCOS group 3%. We calculated a sample of 2780 patients (556 in PCOS group; 2224 in non-PCOS group) which would provide the study with 80% power to detect a difference between groups at a two-sided alpha of 0.05.

To avoid the influences of confounding factors on pregnancy outcomes, these factors including age, infertility type, BMI, as well as number of giving birth and number of pregnancies before IVF/ICSI, underwent PSM. The Logistics regression model was established with the group as a dependent variable to calculate the propensity score. The matching tolerance was 0.02, and sampling without replacement was based on 1:1 scale matching.

Data before and after matching were analyzed using SPSS24.0 software. Measurement data were expressed as mean ± standard deviation (x ± s) or median quartiles. Comparisons between groups was performed by *t* test for the measurement data with normal distribution, and for the measurement data without normal distribution by non-parametric test. Numeration data were expressed as ratio or rate (%), and comparisons between groups was performed by chi-square test. Correlation between various parameters was first performed and the correlation coefficient > 0.8 was regarded as a large impact. And then collinearity diagnostics for all independent variables were done by calculating variance inflation factors (VIF), and VIF ≥ 5 suggested multicollinearity. Univariate analysis and multivariate analysis were performed by binary Logistics regression. Variables with statistical differences in the univariate analysis were included in the multivariate regression equation. Statistical significance was established at *P* < 0.05.

## Results

### Baseline data

There were 12,055 fresh oocyte retrieval cycles (about 2009 cycles per year) between January 2015 and December 2020. In the 12,055 cycle, 2083 were diagnosed as PCOS cycles accounting for 17.28% of the total cycles. Based on the inclusion and exclusion criteria and except non-pregnant cycles and only biochemical pregnancy, remaining clinical pregnancy cycles included 613 PCOS cycle and 2363 non-PCOS cycles. Pregnancy outcomes are shown in Supplementary Fig. 1.

The data from 613 PCOS and 2363 non-PCOS clinical pregnancy cycles underwent PSM, and then 599-paired data were successfully matched. The pregnancy outcomes of the 599-paired PCOS and non-PCOS cycles are shown in Supplementary Fig. 2.

Before PSM, age, infertility type, BMI, AMH, basal FSH, basal LH, basal testosterone, AFC, as well as number of giving birth and number of pregnancies before IVF/ICSI were statistically different between PCOS and non-PCOS clinical pregnancy cycles (all *P* < 0.05). Only age, infertility type, BMI, as well as number of giving birth and number of pregnancies before IVF/ICSI underwent PSM. After PSM, there were no statistical differences in age, infertility type, BMI, as well as number of giving birth and number of pregnancies before IVF/ICSI between PCOS and non-PCOS clinical pregnancy cycles (all *P* > 0.05), but AMH, basal FSH, basal LH, basal testosterone and AFC were still statistically different between the two groups (all *P* < 0.05). Comparisons of baseline data between PCOS and non-PCOS clinical pregnancy cycles before and after PSM are shown in Table 1.

**Table 1:** Comparisons of baseline data between PCOS and non-PCOS clinical pregnancy cycles.

Items	Before PSM		*P*	After PSM		*P*
	PCOS (*n* = 613)	Non-PCOS (*n* = 2363)		PCOS (*n* = 599)	Non-PCOS (*n* = 599)	
Age (year)	29.19 ± 4.01	31.59 ± 4.67	0.000	29.28 ± 4.00	29.46 ± 4.45	0.452
Infertile period (year)	3.90 ± 2.54	3.82 ± 2.90	0.481	3.92 ± 2.56	3.71 ± 2.67	0.176
Primary infertility (n, %)	330 (53.83)	905 (38.30)	0.000	318 (53.09)	311 (51.92)	0.685
Secondary infertility (n, %)	283 (46.17)	1458 (61.70)		281 (46.91)	288 (48.08)	
BMI (kg/m^2^)	24.40 (22.00,27.30)	22.60 (20.60,25.10)	0.000	24.72 ± 3.92	24.53 ± 4.32	0.800
AMH (ng/ml)	7.28 (4.62,10.46)	2.96 (1.84,4.69)	0.000	7.27 (4.62, 10.40)	3.47(2.13, 5.27)	0.000
Basal FSH (mIU/ml)	6.07 (4.89,7.22)	6.70 (5.38,8.30)	0.000	6.11 ± 2.09	6.51 ± 2.40	0.002
Basal LH (mIU/ml)	5.42 (3.48,8.67)	3.97 (2.76,5.49)	0.000	5.35 (3.08, 8.68)	3.80(2.46, 5.49)	0.000
Basal T (ng/ml)	0.57 (0.43,0.73)	0.44 (0.31,0.59)	0.000	0.58 (0.43, 0.74)	0.48(0.35, 0.64)	0.000
AFC	31.88 ± 8.04	18.16 ± 8.40	0.000	31.80 ± 8.00	19.53 ± 8.40	0.000
Number of pregnancy	0.74 ± 1.01	1.18 ± 1.27	0.000	0.75 ± 1.02	0.84 ± 1.14	0.148
Number of giving birth	0.22 ± 0.43	0.33 ± 0.50	0.000	0.22 ± 0.43	0.22 ± 0.42	0.893
Number of abortion	0.03 ± 0.19	0.05 ± 0.24	0.053	0.03 ± 0.19	0.03 ± 0.19	0.633
Starting dose of Gn	192.17 ± 50.26	225.74 ± 52.22	0.000	191.86 ± 50.39	219.98 ± 50.54	0.000
Duration of Gn	12.42 ± 3.67	11.03 ± 2.32	0.000	12.38 ± 3.64	11.27 ± 2.42	0.000
Total amount of Gn	2637.97 ± 1196.53	2609.89 ± 883.62	0.587	2618.16 ± 1186.11	2620.58 ± 897.38	0.968
Number of embryos transferred	1.95 ± 0.35	1.98 ± 0.40	0.120	1.95 ± 0.36	1.96 ± 0.39	0.440

PCOS, polycystic ovary syndrome; PSM, propensity score matching; BMI, body mass index, AMH, Anti-Mullerian hormone; FSH, follicle-stimulating hormone; LH, luteinizing hormone; T, testosterone; Gn, gonadotrophin.

Starting dose of Gn and duration of Gn were statistically different (*P* < 0.05), but total amount of Gn was not statistically different between the PCOS and non-PCOS clinical pregnancy cycles before and after PSM (*P* > 0.05) (Table 1).

### Pregnancy outcomes

Comparisons of pregnancy outcomes between PCOS and non-PCOS clinical pregnancy cycles before and after PSM are shown in Table 2.

**Table 2:** Comparisons of pregnancy outcomes between PCOS and non-PCOS clinical pregnancy cycles.

	Before PSM				After PSM			
	PCOS (*n* = 613)	Non-PCOS (*n* = 2363)	*P*	OR (95%CI)	PCOS (*n* = 599)	Non-PCOS (*n* = 599)	*P*	OR (95%CI)
Early abortion rate (n, %)	59 (9.62)	294 (12.44)	0.058	0.75 (0.56 ~ 1.01)	57 (9.52)	70 (11.69)	0.222	0.80 (0.55 ~ 1.15)
Ectopic pregnancy rate (n, %)	9 (1.47)	46 (1.95)	0.433	0.75 (0.37 ~ 1.54)	9 (1.50)	15 (2.50)	0.216	0.59 (0.26 ~ 1.39)
Multiple pregnancy rate (n, %)	234 (38.17)	800 (33.86)	0.045	1.21 (1.00 ~ 1.45)	231 (38.56)	206 (34.39)	0.133	1.20 (0.95 ~ 1.52)
Overall late abortion rate (n, %)	31 (5.69)	51 (2.52)	0.000	2.33 (1.48 ~ 3.68)	30 (5.63)	17 (3.31)	0.070	1.74 (0.95 ~ 3.20)
Late abortion rate in sin gletons (n,%)	10 (3.12)	28 (2.22)	0.351	1.42 (0.68 ~ 2.94)	9 (2.88)	9 (2.88)	1.000	1.00 (0.39 ~ 2.55)
Late abortion rate in twins (n, %)	21 (9.38)	23 (3.01)	0.000	3.33 (1.81 ~ 6.14)	21 (9.50)	8 (3.96)	0.024	2.55 (1.10 ~ 5.89)
Stillbirth rate (n, %)	4 (0.79)	6 (0.31)	0.133	2.57 (0.07 ~ 9.14)	4 (0.80)	3 (0.60)	0.712	1.33 (0.30 ~ 5.95)
Final live birth ratio (n, %)	505 (82.38)	1946 (82.35)	0.987	1.00 (0.79 ~ 1.27)	494 (82.47)	492 (82.14)	0.940	1.02 (0.76 ~ 1.38)
Pregnancy complication rate (n, %)	6 (0.98)	34 (1.44)	0.378	0.67 (0.28 ~ 1.62)	6 (1.00)	13 (2.17)	0.105	0.46 (0.17 ~ 1.21)

PCOS, polycystic ovary syndrome; PSM, propensity score matching.

Before PSM, multiple pregnancy rate, overall late abortion rate and late abortion rate of twin pregnancy were significantly higher in PCOS clinical pregnancy cycles than in non-PCOS clinical pregnancy cycles (all *P* < 0.05). However, after PSM, only the late abortion rate of twin pregnancy was significantly higher in the PCOS clinical pregnancy cycles (9.50%) than in the non-PCOS clinical pregnancy cycles (3.96%) (*P* < 0.05), other pregnancy outcomes were not statistically different (all* P* > 0.05).

### Etiological distribution of late abortion

According to medical record, the etiology distribution of late abortion in the PCOS and non-PCOS groups after PSM is shown in Table 3. The etiology distribution was not statistically significant both in singletons and twins between the two groups (*P* > 0.05).

**Table 3:** Etiology distribution of late abortion in the PCOS and non-PCOS groups after PSM.

	Singleton pregnancy		Twin pregnancy		Statistic	*P*
	PCOS group (*n* = 9)	Non-PCOS group (*n* = 9)	PCOS group (n = 21)	Non-PCOS group (n = 8)		
Cervical incompetence	0	2	7	3	23.46	0.255
Premature rupture of membranes	2	2	7	2		
Embryo damage	2	2	0	0		
Spontaneous abortion	4	2	4	2		
Infection	0	0	2	1		
Pregnancy-induced hypertension	1	0	0	0		
Uterine contraction	0	0	1	0		
Knot of umbilical cord	0	1	0	0		

PCOS, polycystic ovary syndrome; PSM, propensity score matching.

### Analysis of risk factors for late abortion in twin pregnancy

In the data of twin pregnancy after PSM, the late abortion was used as a dependent variable, and PCOS, age, infertile period, infertility type, BMI, number of embryos transferred as well as numbers of pregnancy, giving birth and abortion before IVF/ICSI were used as independent variables. Correlation between various parameters indicated that all correlation coefficients were less than 0.8 (Supplementary Table 1) and the collinearity diagnostics for all independent variables displayed all VIF < 5 (Supplementary Table 2). Univariate analysis was performed by Logistics regression.

The variables with statistical differences in the univariate analysis underwent multivariate analysis. The results of univariate and multivariate analyses are shown in Table 4. PCOS and obesity (pregnancy-assisted BMI ≥ 28) were risk factors for late abortion in twin pregnancy (*P* < 0.05). In twin pregnancy, the late abortion risk significantly increased in the PCOS patient as compared with non-PCOS patients (OR: 2.59, 95%CI 1.11–6.03, *P* < 0.05), as well as in the patients with obesity (BMI ≥ 28) as compared with the patients with normal BMI (OR: 4.17, 95%CI 1.59–10.90, *P* < 0.05).

**Table 4:** Analysis of risk factors for late abortion in twin pregnancy (Based on the data after PSM for clinical pregnancy).

	Univariate regression		Multivariate regression	
	*P*	OR (95%CI)	*P*	aOR (95%CI)
PCOS				
No	Reference		Reference	
Yes	0.029	2.55 (1.10 ~ 5.89)	0.028	2.59 (1.11 ~ 6.03)
Age				
< 35 years	Reference		–	
≥ 35 years	0.781	0.81 (0.18 ~ 3.56)		
Infertile period	0.630	1.04 (0.90 ~ 1.19)	–	
Infertile type				
Primary infertility	Reference		–	
Secondary infertility	0.496	1.30 (0.61 ~ 2.77)		
BMI				
Normal (BMI < 24)	Reference		Reference	
Overweight (24 ≤ BMI < 28)	0.267	1.72 (0.67 ~ 4.46)	0.250	1.75 (0.67 ~ 4.58)
Obesity (BMI ≥ 28)	0.004	4.10 (1.58 ~ 10.65)	0.004	4.17 (1.59 ~ 10.90)
Number of pregnancies	0.971	1.01 (0.71 ~ 1.44)	–	
Number of giving birth	0.120	0.39 (0.12 ~ 1.28)	–	
Number of abortions	0.625	0.50 (0.03 ~ 7.84)	–	
Number of embryos transferred	0.578	0.59 (0.09 ~ 3.74)	–	

PCOS, polycystic ovary syndrome; PSM, propensity score matching; BMI, body mass index.

## Discussion

This study indicated that after PSM for baseline data, the early and late abortion rates were not statistically different between the PCOS group and the non-PCOS group. The further stratified analysis displayed that only in twin pregnancy, the late abortion rate was significantly higher in the PCOS group than in the non-PCOS group. Logistics regression exhibited that PCOS and obesity [pregnancy-assisted BMI ≥ 28 kg/m^2^] were risk factors for late abortion in twin pregnancy.

Hyperandrogenemia, obesity, insulin resistance and metabolic abnormalities present in PCOS patients all may lead to an increased risk of complications during gestational period^6–9^. There have been different reports on whether the abortion rate increases in PCOS patients^9,11,12,15^. In most studies, the abortion rate is usually used as a pregnancy outcome, rather than is clearly divided into early and late abortions. In China, the abortion occurring between 4 and 12 gestational weeks is defined as early abortion, and occurring between 12 and 28 gestational weeks as late abortion. It has been reported that the risk of early abortion does not increase in PCOS patients, and the increased abortion rate is mainly due to the elevation of late abortion rate^16^. The data before PSM in this study also obtained the similar result that there was no statistical difference in the early abortion rate between the PCOS group and non-PCOS group, but the overall late abortion rate was significantly higher in the PCOS group (5.69%) as compared with the non-PCOS group (2.52%). To avoid the influences of confounding factors on pregnancy outcomes, PSM was used to balance baseline data between the two groups in this study, making the comparison of pregnancy outcomes more credible. Post-PSM baseline data showed that the overall late abortion rate was not statistically different between the PCOS group and non-PCOS group, but the late abortion rate in twin pregnancy was significantly higher in the PCOS group (9.50%) than in the non-PCOS group (3.56%). The data before and after PSM indicated different comparison results between the PCOS group and non-PCOS group about the overall late abortion rate. This may be related to the confounding bias of clinical factors. Some studies have shown that obesity is related to abortion^16–19^. Therefore, the late abortion rate is usually high in PCOS group because PCOS patients is frequently associated with overweight or obesity. In this study, no statistical difference in the overall late abortion rate between the PCOS group and non-PCOS group after PSM may be related to balancing the baseline data. Joham et al.^19^ believed that PCOS could not independently predict abortion risk, but overweight and obesity were independently associated with abortion.

Embryo, endometrium and the interaction between both are three important factors affecting pregnancy. Alterations in oocyte competence are considered potential causative factors for subfertility in women with PCOS^20^. Clinical characteristics related to the syndrome, such as hyperandrogenemia and obesity, may contribute to dysregulation of endometrial expression of sex hormone receptors and co-receptors, increase endometrial insulin-resistance with impaired glucose transport and utilization, and result in chronic low-grade inflammation, immune dysfunction, altered uterine vascularity, abnormal endometrial gene expression and cellular abnormalities in women with PCOS^21^. The phenotypes of PCOS vary with the severity of these clinical features. This may lead to inconsistent conclusions among different studies.

In this study, although the number of embryos transferred and multiple pregnancy rate were similar between the PCOS group and non-PCOS group after PSM, the late abortion rate of twin pregnancy was significantly higher in the PCOS group (9.50%) than in the non-PCOS group (3.96%). PCOS patients have abnormal metabolism, such as luteum hormone elevation, high testosterone, obesity and insulin resistance. Androgen regulates cervical collagen fiber tissue during pregnancy^22^, affects the cervical remodeling and promotes cervical ripening. Therefore, in most PCOS patients, uterine is small with cervical dysplasia and cervical incompetence. In twin pregnancy, the uterine pressure increases and the cervix bears the greater gravity effect, which aggravates pregnancy burden^23^ and makes pregnancy outcomes worse. Assisted reproductive technology is currently a main cause of iatrogenic multiple pregnancy. Adverse obstetric outcomes in PCOS patients may be associated with multiple pregnancy^24^. In recent years, the reproductive field has been constantly advocating to reduce the number of embryos transferred to decrease the rate of multiple pregnancy, but this has not been strictly emphasized on PCOS patients. Based on our results, we recommend that single embryo transfer should be included into the transplantation strategy for PCOS patients.

In this study, Logistics regression showed that PCOS and obesity were risk factors for late abortion in twin pregnancy. Obesity is a common comorbidity in PCOS patients^8^. Overweight or obesity can exacerbate metabolic abnormalities in PCOS patients. For example, obesity aggravates insulin resistance and hyperinsulinemia, which in turn increases fat formation. Obesity increases the sensitivity of follicle membrane cells to luteinizing hormone and upregulates ovarian androgen production, which aggravates endocrine disorder of PCOS^25^. Obesity may increase the risk of abortion^16–19^. In PCOS patients, clinical features such as insulin resistance, metabolic abnormalities and chronic low-grade inflammation as well as comorbidities such as obesity and diabetes affect trophoblast invasion and placenta formation, increasing the risk of complications for maternal and infant during pregnancy^8,26^. Coexistence of multiple risk factors may allow the risk of pregnancy complications to reach a clinical threshold^26^.

In this study, we collected the causes of late abortion mainly included cervical incompetence, premature rupture of membranes, embryo damage, spontaneous abortion, etc. Etiological statistics on late abortion in PCOS patients receiving IVF/ ICSI have not been reported. This study indicated no statistical difference in the etiological distribution of late abortion between PCOS and non-PCOS groups, suggesting that PCOS alone is not a specific cause of late abortion, late abortion is caused by PCOS combined with multiple adverse factors.

The advantages of this study were (1) a retrospective cohort study from real-world data; (2) application of PSM in selecting data; (3) observing early and late abortions; (4) observing abortion rates in singletons and twins; (5) observing etiological distribution of late abortion; and (6) analyzing risk factors of late abortion. However, there were some limitations in this study. Firstly, PSM can effectively reduce the confounding bias, but PSM only can match known factors, and not completely exclude unbalanced factors^27^. Secondly, some important factors, such as blood pressure, blood sugar, hormone level and cervical length during pregnancy were absent in some patients due to retrospective study. Thirdly, this study was based on Chinese PCOS patients receiving IVF, so the results obtained from this study were not suitable to all PCOS patients and other racial individuals. The conclusion of this study remains to be further confirmed by large-sample and multi-center prospective studies. We will cooperate with perinatal health care departments to establish a sound whole-process health management system for PCOS patients.

## Conclusion

PCOS does not significantly affect early and overall late abortion rates. However, in twin pregnancy, PCOS and obese patients have higher late abortion rate. PCOS patients still require intensive monitoring and strict weight control after assisted reproduction success. It is not easy to implement weight loss during pregnancy, so patients should lose weight by at least 5–10% before pregnancy^10^. Single embryo transfer may be included into the transplantation strategy for PCOS patients.

## Supplementary Information

Supplementary Information 1.

Supplementary Information 2.

Supplementary Information 3.

Supplementary Information 4.

## Data Availability

The datasets used and/or analyzed during the current study available from the corresponding author on reasonable request.

## References

[1] Teede H, Deeks A, Moran L (2010). Polycystic ovary syndrome: A complex condition with psychological, reproductive and metabolic manifestations that impacts on health across the lifespan. BMC Med..

[2] Azziz R, Carmina E, Chen Z, Dunaif A, Laven JS, Legro RS (2016). Polycystic ovary syndrome. Nat. Rev. Dis. Primers..

[3] Thessaloniki ESHRE/ASRM-Sponsored PCOS Consensus Workshop Group (2008). Consensus on infertility treatment related to polycystic ovary syndrome. Hum. Reprod..

[4] Balen AH, Morley LC, Misso M, Franks S, Legro RS, Wijeyaratne CN (2016). The management of anovulatory infertility in women with polycystic ovary syndrome: An analysis of the evidence to support the development of global WHO guidance. Hum. Reprod. Update.

[5] Rotterdam ESHRE/ASRM-Sponsored PCOS Consensus Workshop Group (2004). Revised 2003 consensus on diagnostic criteria and long-term health risks related to polycystic ovary syndrome. Fertil. Steril..

[6] Boomsma CM, Eijkemans MJ, Hughes EG, Visser GH, Fauser BC, Macklon NS (2006). A meta-analysis of pregnancy outcomes in women with polycystic ovary syndrome. Hum. Reprod. Update.

[7] Bahri Khomami M, Joham AE, Boyle JA, Piltonen T, Silagy M, Arora C (2019). Increased maternal pregnancy complications in polycystic ovary syndrome appear to be independent of obesity-A systematic review, meta-analysis, and meta-regression. Obes. Rev..

[8] Palomba S, de Wilde MA, Falbo A, Koster MP, La Sala GB, Fauser BC (2015). Pregnancy complications in women with polycystic ovary syndrome. Hum. Reprod. Update.

[9] Yu HF, Chen HS, Rao DP, Gong J (2016). Association between polycystic ovary syndrome and the risk of pregnancy complications: A PRISMA-compliant systematic review and meta-analysis. Medicine (Baltimore).

[10] Valent AM, Barbour LA (2021). Management of women with polycystic ovary syndrome during pregnancy. Endocrinol. Metab. Clin. North Am..

[11] Sha T, Wang X, Cheng W, Yan Y (2019). A meta-analysis of pregnancy-related outcomes and complications in women with polycystic ovary syndrome undergoing IVF. Reprod. Biomed. Online.

[12] Li HW, Lee VC, Lau EY, Yeung WS, Ho PC, Ng EH (2014). Cumulative live-birth rate in women with polycystic ovary syndrome or isolated polycystic ovaries undergoing in-vitro fertilisation treatment. J. Assist. Reprod. Genet..

[13] Tang K, Wu L, Luo Y, Gong B (2021). In vitro fertilization outcomes in women with polycystic ovary syndrome: A meta-analysis. Eur. J. Obstet. Gynecol. Reprod. Biol..

[14] Lan-lan C, Li T, Zong-gang F, Li-jing W (2021). Effect of atosiban on IVF fresh transfer cycle. Chin. J. Reprod. Contracept..

[15] Xu-jing G (2020). Effects of phloroglucinol on transfer outcome of in vitro fertilization-embryo transfer. Chin. J. Reprod. Contracept..

[16] Cai H, Mol BW, Gordts S, Wang H, Wang T, Li N (2021). Early and late pregnancy loss in women with polycystic ovary syndrome undergoing IVF/ICSI treatment: A retrospective cohort analysis of 21 820 pregnancies. BJOG.

[17] Yang AM, Xu X, Han Y, Wei JJ, Hao GM (2021). Risk factors for different types of pregnancy losses: Analysis of 15,210 pregnancies after embryo transfer. Front. Endocrinol. (Lausanne).

[18] Supramaniam PR, Mittal M, McVeigh E, Lim LN (2018). The correlation between raised body mass index and assisted reproductive treatment outcomes: A systematic review and meta-analysis of the evidence. Reprod. Health.

[19] Romanski PA, Bortoletto P, Magaoay B, Chung A, Rosenwaks Z, Spandorfer SD (2021). Live birth outcomes in infertile patients with class III and class IV obesity following fresh embryo transfer. J. Assist. Reprod. Genet..

[20] Palomba S, Daolio J, La Sala GB (2017). Oocyte competence in women with polycystic ovary syndrome. Trends Endocrinol. Metab..

[21] Palomba S, Piltonen TT, Giudice LC (2021). Endometrial function in women with polycystic ovary syndrome: A comprehensive review. Hum. Reprod. Update.

[22] Makieva S, Saunders PT, Norman JE (2014). Androgens in pregnancy: Roles in parturition. Hum. Reprod. Update.

[23] Santana DS, Cecatti JG, Surita FG, Silveira C, Costa ML, Souza JP (2016). Twin pregnancy and severe maternal outcomes: The World Health Organization multicountry survey on maternal and newborn health. Obstet. Gynecol..

[24] Mikola M, Hiilesmaa V, Halttunen M, Suhonen L, Tiitinen A (2001). Obstetric outcome in women with polycystic ovarian syndrome. Hum. Reprod..

[25] Glueck CJ, Goldenberg N (2019). Characteristics of obesity in polycystic ovary syndrome: Etiology, treatment, and genetics. Metabolism.

[26] Palomba S (2021). Is fertility reduced in ovulatory women with polycystic ovary syndrome? An opinion paper. Hum. Reprod..

[27] Wang J (2021). To use or not to use propensity score matching?. Pharm. Stat..

