# Supplementary material for: The influence of polycystic ovary syndrome on abortion rate after in vitro fertilization/intracytoplasmic sperm injection fresh cycle pregnancy
Source: Sci Rep. 2023 Apr 12;13:5978. doi: 10.1038/s41598-023-32988-5 (PMC10097689; doi:10.1038/s41598-023-32988-5)
Supplement: Supplementary file 1 — Supplementary Information 1. [file 41598_2023_32988_MOESM1_ESM.docx]

Oocyte retrieval cycles 12055

PCOS cycles 1885

Non-PCOS cycles 8930

Transfer cycles 1011

Transfer cycles 4489

Clinical pregnancy 613

Clinical pregnancy 2363

Ongoing pregnancy 545

Ongoing pregnancy 2023

Live birth 505

Live birth 1946

Unpregnancy 355, only biochemical pregnancy 43

Early abortion 59, ectopic pregnancy 9

Later abortion 31, induced labour 5, stillbirth 4

Unpregnancy 1955, only biochemical pregnancy 171

Early abortion 294, ectopic pregnancy 46

Later abortion 51, induced labour 20, stillbirth 6

Exclusion criteria

**Supplementary Figure 1** Pregnancy outcomes in PCOS and non-PCOS cycles

Notes: PCOS: polycystic ovary syndrome
