# Supplementary material for: The influence of polycystic ovary syndrome on abortion rate after in vitro fertilization/intracytoplasmic sperm injection fresh cycle pregnancy
Source: Sci Rep. 2023 Apr 12;13:5978. doi: 10.1038/s41598-023-32988-5 (PMC10097689; doi:10.1038/s41598-023-32988-5)
Supplement: Supplementary file 2 — Supplementary Information 2. [file 41598_2023_32988_MOESM2_ESM.docx]

PCOS group

599 cycles

Non-PCOS group 599 cycles

Clinical pregnancy after PSM

Early abortion 57

异位妊娠9

Early abortion 70,

ectopic pregnancy 15

Ongoing pregnancy 533

Ongoing pregnancy 514

Live birth 494

Live birth 492

Later abortion 30,

induced labour 5,

stillbirth 4

Later abortion 17, induced labour 2, stillbirth 3

**Supplementary Figure 2** Pregnancy outcomes in both groups after PSM

Notes: PCOS: polycystic ovary syndrome; PSM: propensity score matching
