# Supplementary material for: The influence of polycystic ovary syndrome on abortion rate after in vitro fertilization/intracytoplasmic sperm injection fresh cycle pregnancy
Source: Sci Rep. 2023 Apr 12;13:5978. doi: 10.1038/s41598-023-32988-5 (PMC10097689; doi:10.1038/s41598-023-32988-5)
Supplement: Supplementary file 3 — Supplementary Information 3. [file 41598_2023_32988_MOESM3_ESM.docx]

**Supplementary Table 1** Correlation between various parameters

|  | | PCOS | Age | Infertile period | Infertile type | BMI | Number of pregnancies | Number of giving birth | Number of abortions | Number of embryos transferred |
| --- | --- | --- | --- | --- | --- | --- | --- | --- | --- | --- |
| PCOS | CC | 1 | 0.047 | 0.061 | 0.097 | -0.001 | 0.035 | 0.079 | 0.041 | -0.040 |
|  | *P* |  | 0.339 | 0.210 | 0.047 | 0.978 | 0.469 | 0.103 | 0.398 | 0.415 |
| Age | CC | 0.047 | 1 | 0.146 | 0.225 | -0.077 | 0.185 | 0.216 | -0.006 | 0.382 |
|  | *P* | 0.339 |  | 0.003 | 0.000 | 0.113 | 0.000 | 0.000 | 0.905 | 0.000 |
| Infertile period | CC | 0.061 | 0.146 | 1 | -0.138 | 0.134 | -0.216 | 0.011 | 0.016 | 0.022 |
|  | *P* | 0.210 | 0.003 |  | 0.005 | 0.006 | 0.000 | 0.819 | 0.750 | 0.646 |
| Infertile type | CC | 0.097 | 0.225 | -0.138 | 1 | -0.098 | 0.775 | 0.573 | 0.189 | 0.166 |
|  | *P* | 0.047 | 0.000 | 0.005 |  | 0.045 | 0.000 | 0.000 | 0.000 | 0.001 |
| BMI | CC | -0.001 | -0.077 | 0.134 | -0.098 | 1 | -0.095 | -0.047 | -0.048 | -0.060 |
|  | *P* | 0.978 | 0.113 | 0.006 | 0.045 |  | 0.050 | 0.331 | 0.330 | 0.218 |
| Number of pregnancies | CC | 0.035 | 0.185 | -0.216 | 0.775 | -0.095 | 1 | 0.540 | 0.299 | 0.166 |
|  | *P* | 0.469 | 0.000 | 0.000 | 0.000 | 0.050 |  | 0.000 | 0.000 | 0.001 |
| Number of giving birth | CC | 0.079 | 0.216 | 0.011 | 0.573 | -0.047 | 0.540 | 1 | 0.163 | 0.072 |
|  | *P* | 0.103 | 0.000 | 0.819 | 0.000 | 0.331 | 0.000 |  | 0.001 | 0.140 |
| Number of abortions | CC | 0.041 | -0.006 | 0.016 | 0.189 | -0.048 | 0.299 | 0.163 | 1 | 0.009 |
|  | *P* | 0.398 | 0.905 | 0.750 | 0.000 | 0.330 | 0.000 | 0.001 |  | 0.861 |
| Number of embryos transferred | CC | -0.040 | 0.382 | 0.022 | 0.166 | -0.060 | 0.166 | 0.072 | 0.009 | 1 |
|  | *P* | 0.415 | 0.000 | 0.646 | 0.001 | 0.218 | 0.001 | 0.140 | 0.861 |  |
|  |  |  |  |  |  |  |  |  |  |  |
