# Supplementary material for: The influence of polycystic ovary syndrome on abortion rate after in vitro fertilization/intracytoplasmic sperm injection fresh cycle pregnancy
Source: Sci Rep. 2023 Apr 12;13:5978. doi: 10.1038/s41598-023-32988-5 (PMC10097689; doi:10.1038/s41598-023-32988-5)
Supplement: Supplementary file 4 — Supplementary Information 4. [file 41598_2023_32988_MOESM4_ESM.docx]

| **Supplementary Table 2** Collinearity diagnostics for all independent variables | | |
| --- | --- | --- |
|  | Tolerance | VIF |
| PCOS | 0.974 | 1.026 |
| Age | 0.774 | 1.291 |
| Infertile period | 0.872 | 1.146 |
| Infertile type | 0.355 | 2.815 |
| BMI | 0.966 | 1.035 |
| Number of pregnancies | 0.343 | 2.919 |
| Number of giving birth | 0.622 | 1.609 |
| Number of abortions | 0.891 | 1.123 |
| Number of embryos transferred | 0.828 | 1.208 |

Notes: PCOS: polycystic ovary syndrome; VIF: variance inflation factors; BMI: body mass index
